# Impact of the Nuclear Envelope on Malignant Transformation, Motility, and Survival of Lung Cancer Cells

**DOI:** 10.1002/advs.202102757

**Published:** 2021-10-17

**Authors:** Sílvio Terra Stefanello, Isabelle Luchtefeld, Ivan Liashkovich, Zoltan Pethö, Ihab Azzam, Etmar Bulk, Gonzalo Rosso, Lilly Döhlinger, Bettina Hesse, Andrea Oeckinghaus, Victor Shahin

**Affiliations:** ^1^ Institute of Physiology II University of Münster Robert‐Koch‐Str. 27b Münster 48149 Germany; ^2^ Institute of Immunology University of Münster Röntgen‐Str. 21 Münster 48149 Germany; ^3^ Institute of Molecular Tumor Biology University of Münster Robert‐Koch‐Str. 43 Münster 48149 Germany

**Keywords:** cancer, cellular physiology and biophysics, nanomedicine, nuclear envelope, nuclear pores

## Abstract

Nuclear pore complexes (NPCs) selectively mediate all nucleocytoplasmic transport and engage in fundamental cell‐physiological processes. It is hypothesized that NPCs are critical for malignant transformation and survival of lung cancer cells, and test the hypothesis in lowly and highly metastatic non‐small human lung cancer cells (NSCLCs). It is shown that malignant transformation is paralleled by an increased NPCs density, and a balanced pathological weakening of the physiological stringency of the NPC barrier. Pharmacological interference using barrier‐breaking compounds collapses the stringency. Concomitantly, it induces drastic overall structural changes of NSCLCs, terminating their migration. Moreover, the degree of malignancy is found to be paralleled by substantially decreased lamin A/C levels. The latter provides crucial structural and mechanical stability to the nucleus, and interacts with NPCs, cytoskeleton, and nucleoskeleton for cell maintenance, survival, and motility. The recent study reveals the physiological importance of the NPC barrier stringency for mechanical and structural resilience of normal cell nuclei. Hence, reduced lamin A/C levels in conjunction with controlled pathological weakening of the NPC barrier stringency may facilitate deformability of NSCLCs during the metastasis steps. Modulation of the NPC barrier presents a potential strategy for suppressing the malignant phenotype or enhancing the effectiveness of currently existing chemotherapeutics.

## Graphical abstract

Malignant transformation of aggressive lung cancer cells is associated with pathophysiological alteration of the nuclear envelope and the physiological stringency of the selective barrier function of nuclear pore complexes (NPCs). Pharmacological disruption of the NPC barrier instantly gives rise to both drastic structural changes of cancer cells and loss of their motility and migratory behavior.

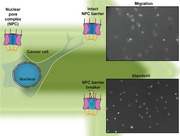

## 1 Introduction

Nuclear pore complexes (NPCs) span the double‐membraned nuclear envelope of eukaryotic cells at regular distances. Their total numbers vary from several hundreds to tens of millions in different cell types and can change dynamically depending on the life cycle, physiological and pathophysiological states amongst others.^[^ ^1^, ^2^, ^3^ ^]^ The NPC is a sophisticated supramolecular cylindrical structure with a rotational octagonal symmetry,^[^ ^4^ ^]^ built from multiple copies of ≈30 different proteins, termed nucleoporins (Nups).^[^ ^5^ ^]^ Two thirds of the Nups build the NPC core scaffold and anchor it within the nuclear envelope while the remaining third generates the selective NPC transport barrier amongst others.^[^ ^6^, ^7^ ^]^ Transport through the NPC proceeds through its central channel that is occupied by Nups exhibiting peculiar biochemical and biophysical properties. They are unstructured, flexible, and highly dynamic and are rich in hydrophobic motifs consisting of varied phenylalanine (F) and glycine (G) repeats that are in turn connected with spacers rich in polar amino acids.^[^ ^8^ ^]^ FG‐Nups provide the NPC with transport selectivity by specific interaction with import and export receptors bearing cargos with identifiable import/export signals. Receptor‐mediated transport can accommodate cargos up to megadalton sizes or ≈39 nm, consistent with the diameter of the NPC channel at its narrowest part.^[^ ^9^ ^]^ At the same time FG‐Nups form a physical barrier inside the NPC channel which restricts unselective transport to a sharp threshold of ≈5 nm or ≈40 kDa.^[^ ^5^ ^]^ In addition to mediating the canonical nucleocytoplasmic transport function of NPCs, several FG‐Nups are potent gene transcription regulators which commute dynamically between the NPC and the nucleus^[^ ^10^ ^]^ to play key roles in development, differentiation, maintenance, and survival of cells.^[^ ^11^, ^12^ ^]^ Alteration of their physiological expression levels is linked to cancer onset and progression,^[^ ^13^, ^14^, ^15^, ^16^, ^17^, ^18^ ^]^ but the underlying mechanisms remain to be explored. There is overwhelming evidence for the crucial roles of alteration of the properties of the cell nucleus and nuclear envelope environment in promoting malignant transformation of cancer cells.^[^ ^13^, ^19^, ^20^, ^21^ ^]^ However, the specific involvement of the NPC barrier function remains barely explored. The vast majority of publications involving NPCs in malignant transformation of cancer cells deal with the roles of certain NPC proteins as transcriptional regulators, while barely any studies specifically address the possible alteration of the selective NPC barrier despite its fundamental relevance for cancer cells survival and therapy.^[^ ^22^ ^]^ Structure and organization of the nucleus, as well as physical cues, interactions, and mechanical forces promoting malignant transformation and metastasis are addressed in comprehensive reviews and citations therein.^[^ ^19^, ^20^, ^23^, ^24^ ^]^ Chow et al., review alterations in the nuclear envelope environment associated with cancer.^[^ ^13^ ^]^ Vargas et al., show transient nuclear envelope rupturing during interphase in human cancer cells.^[^ ^25^ ^]^ Hatch et al., demonstrate catastrophic nuclear envelope collapse in cancer cell micronuclei.^[^ ^26^ ^]^ Denais et al., show nuclear envelope rupture and repair during cancer cell migration.^[^ ^21^ ^]^ Nuclear deformation causes localized loss of nuclear envelope integrity, which leads to uncontrolled exchange of nucleocytoplasmic content, herniation of chromatin across the nuclear envelope, and DNA damage.^[^ ^21^ ^]^ Mohamed et al., reveal loss of mechanical resilience of NPCs in colorectal cancer cells.^[^ ^27^ ^]^ Recent study by McCloskey et al., reports that experimental depletion of the NPC basket protein Tpr dramatically increases the total NPC number in various cell types.^[^ ^1^ ^]^ They suggest the extension of their findings to cancer cells and contemplate that tweaking NPC numbers in cancer cells may usher in the design of novel cancer therapies. Emerging evidence suggests that besides hematological cancers, Nups act as tumor drivers of major non‐hematological malignancies such as carcinomas of skin, breast, lung, pancreas, prostate, and colon. Hence, nucleoporins are emerging as novel therapeutic targets in human tumors.^[^ ^28^ ^]^ As obvious from the aforementioned publications, the specific and direct involvement of the physiological NPC barrier function remains barely explored if at all. Our recent study introduces novel and crucial role of the selective nucleocytoplasmic NPC barrier function in maintaining nuclear mechanics and eventually structure too in normal cells.^[^ ^29^ ^]^ With respect to the critical physiological roles of nuclear structure and mechanics in maintenance and survival of normal cells, pathophysiological alteration in the newly revealed NPC barrier function is most likely to be associated with transformation of normal cells to cancer cells. In the present work, we demonstrate that malignant transformation of lung cancer cells is closely associated with transformation of the NPC barrier. In addition, pharmacological interference with the barrier has direct implications for the survival of cancer cells.

## 2 Results and Discussion

**Figure 1:**
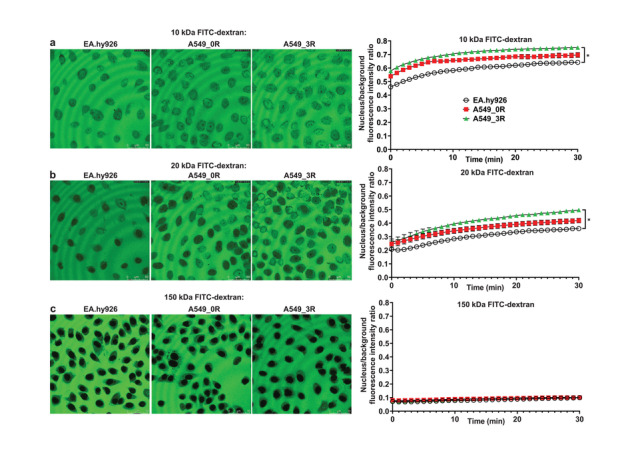
Functional state of the NPC permeability barrier in non‐cancer (EA.hy926) versus cancer cells (A549_0R and A549_3R). a–c left) Confocal microscopy imaging of the three cell lines EA.hy926 (non‐cancerous), A549_0R (cancerous, low malignancy), and A549_3R (high malignancy) show that the nuclei of the cells with the highly aggressive phenotype are characterized by a reduced stringency of the nucleocytoplasmic permeability barrier, tested with fluorescent tracer of different sizes (FITC‐dextran 10, 20 and 150 kDa; 150 kDa serves as functional integrity marker of the nuclear envelope). a,b,c right) The corresponding graphs of (a,b,c left) respectively, show the kinetics of influx of the FITC‐dextrans into the nuclei of the three cell lines, and demonstrate that influx rates correlate with malignancy. Data are shown as the mean ± SEM. Asterisks indicate statistically significant differences (*P* < 0,05, Two Way ANOVA followed by Tukey's Multiple Comparison test). *N* = 10 and ≥400 cells analyzed in each experimental condition. Images are 250 µm × 250 µm each.

The remarkable plasticity of NPCs enables them to undergo changes in their number, structure, function, and composition in response to physiological cues.^[^ ^2^, ^11^ ^]^ The redistribution of oncogene products and tumor suppressors between the cytosol and the nucleus of cancer cells^[^ ^30^ ^]^ hints at a possible alteration of the primary transport and/or barrier function of the NPCs. We set out to study potential changes of NPCs in lowly metastatic (A549_0R) and highly metastatic (A549_3R) non‐small cell lung cancer (NSCLC) cell lines,^[^ ^31^ ^]^ in comparison to non‐cancer cells. Lung cancer is worldwide by far the most common cause of cancer deaths, accounting for almost 25% of all cases, resulting in well over 1.6 million deaths each year,^[^ ^32^, ^33^ ^]^ and the histological subtypes NSCLC are the most common accounting for ≈85%.^[^ ^32^ ^]^ In spite of significant clinical advances made in cancer research over the past years, the 5‐year relative survival rate of patients with metastatic NSCLC remains extremely low at 5%.^[^ ^33^ ^]^ Continued research of the pathophysiological mechanisms underlying the molecularly heterogeneous NSCLC is crucial to increasing the effectiveness of therapies and advancing the devastating prognosis.^[^ ^32^ ^]^ The cell lines we used are proper in vitro models for NSCLC. The lowly metastatic and commercially available A549 ((ATCC CCL‐185)), is human epithelial carcinoma cell line derived from explanted cultures of human lung cancer tissue of a 58 year old Caucasian patient,^[^ ^34^ ^]^ and is widely used for basic cancer research and drug discovery. They retain cancerous characteristics associated with their original tumor.^[^ ^34^ ^]^ In the present work, we refer to A549 as A549_0R. A549_3R are highly aggressive NSCLCS generated from the parental A549_0R cell line in an in vivo selection approach in mice, following three rounds (R) of selection upon injection of A549_0R into the tail vein of mice, as described in the original publication.^[^ ^31^ ^]^ The high metastatic potential of A549_3R was demonstrated in vivo in NOD/SCID mice following intravenous injection.^[^ ^31^ ^]^ Further in vitro investigations showed that A549_3R exhibited additional features linked to their high metastatic potential, including enhanced clonogenic growth and increased mutation rate.^[^ ^31^ ^]^ Consistently, our previous publication demonstrates that migration and proliferation rate of A549_3R cells in vitro are enhanced.^[^ ^35^ ^]^ As control for non‐cancer cells, we utilized the human cell line EA.hy926. This cell line was obtained by fusing the stable human cell line A549 with primary human umbilical vein endothelial cells.^[^ ^36^ ^]^ The resulting immortalized EA.hy926 cell line preserves the specific cellular physiological properties of the primary cell line, for instance the release of Weibel–Palade bodies and factor VIII‐related antigen. It also exhibits tissue‐specific organelles, characteristics of differentiated endothelial cell functions such as angiogenesis, homeostasis/thrombosis, blood pressure, and inflammation, which is why it widely serves as valuable in vitro biomedical model for vascular endothelial cell research.^[^ ^36^, ^37^ ^]^ We started our investigations by examining passive NPC permeability, which is a measure of the NPC barrier stringency, in control versus A540_0R and A549_3R cells, using fluorescein isothiocyanate (FITC) dextrans of different sizes. The utilized experimental approach is based on the well‐established digitonin‐permeabilized cell assay;^[^ ^38^ ^]^ each experimental condition is repeated five times or more and the total number of analyzed cells is at least 200 in each individual condition. The choice of the molecular sizes 10, 20, and 150 kDa is based on the stringency and functional integrity of the NPC permeability barrier, which was experimentally determined to have an upper cut‐off < 40 kDa for FITC‐dextran;^[^ ^39^ ^]^ NPCs should permit passive diffusion of 10 and 20 kDa dextrans at decreasing size‐dependent rates, whereas 150 kDa serves as an integrity marker for both the NPC and nuclear envelope.^[^ ^38^, ^39^ ^]^ The results of the NPC barrier stringency measurements are summarized in **Figure** **1**.

All tested cells exhibit high passive NPC permeability for 10 kDa FITC‐dextran. However, the relative permeability levels of A549_0 and A549_3R for 10 kDa FITC‐dextran at the start of the measurement are 14.8 ± 1.8% and 20 ± 1.5% higher than control, respectively. The differences in the levels are largely maintained well after an equilibrium is reached. The permeability levels also increase significantly from control to A549_0 to A549_3R cells for 20 kDa FITC‐dextran. However, unlike 10 kDa FITC‐dextran, the relative permeabilities of the tested cells for 20 kDa FITC‐dextran start out at almost the same levels, which then increase from control to A549_0R to A549_3R. At the end of the measurement, the relative permeability levels of A549_0R and A549_3R are 13 ± 2.7% and 26 ± 2.4% higher than control, respectively. All analyzed cells fully exclude 150 kDa FITC‐dextran from nuclear entry. This demonstrates the integrity of the nuclear envelope permeability barrier^[^ ^38^ ^]^ in all analyzed non‐cancer and cancer cells and rules out a possible presence of membrane ruptures or NPC barrier leakiness which would arise from cell cycle‐dependent changes in the overall structure of the nuclear envelope.^[^ ^40^ ^]^

**Figure 2:**
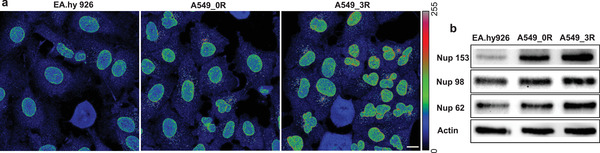
NPCs density in non‐cancer (EA.hy926) versus cancer cells (A549_0R and A549_3R). a) Immunofluorescence staining with mAb414 antibody, which recognizes at least four different FG‐nups.^[^ ^41^, ^42^ ^]^ b) Western blots of the expression levels of Nups 62, Nup98, and Nup153. Malignant transformation of cancer cells is associated with increased NPC density. Scale bar is 10 µm, and *N* = 3 in both experiments.

The aforementioned measurements show that the NPC barrier stringency in lung cancer cells is weakened compared to control non‐cancer cells, and that the transformation from lowly metastatic to highly metastatic stage is associated with gradual weakening of the NPC barrier stringency. The observations indicate that in addition to weakened barrier stringency, an increase in total number of NPCs in the nuclear envelope gives rise to increased nuclear envelope permeability, as cancer cells progress from lowly metastatic to highly metastatic stage. This suggestion is supported by immunostaining and western blots experiments shown in **Figure** **2**.

A monoclonal anti‐Nups antibody (mAb414), which recognizes at least four different FG‐Nups,^[^ ^41^, ^42^ ^]^ is utilized for the detection of NPCs in nuclear envelopes of non‐cancer and cancer cells. Confocal microscopy images in Figure 2a reveal that the brightness of the nuclear envelopes, as a function of NPC density, rises gradually from non‐cancer (control), through A549_0R to A549_3R cells (*N* = 3). Consistently, western blotting experiments (*N* = 3) show progressive increase of NPC proteins (tested for major FG‐Nups 62, 98, and 153) from control, through A549_0R to A549_3R cells (Figure 2b). We therefore conclude that the increase of passive nuclear envelope permeability in cancer cells results partly from increased NPC density in nuclear envelopes, and reason that this might be a consequence of increasing metabolic demands on transformation from non‐cancerous to cancerous types. Figure 2a also shows that nuclei of cancer cells are substantially deformed compared to the homogenous nuclei of non‐cancer cells, which hints at increased compliance of malignant cell nuclei. With respect to the central roles of the nuclear lamina proteins lamins A/C in the overall biomechanical and structural behavior of nuclei,^[^ ^43^, ^44^ ^]^ we wondered whether alteration of their physiological levels is associated with malignant transformation of cells.

**Figure 3:**
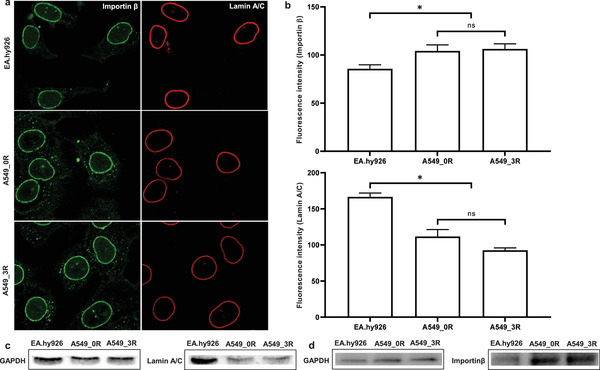
Lamin A/C levels in non‐cancer (EA.hy926) versus cancer cells (A549_0R and A549_3R). a) Immunofluorescence staining of NPCs (importin *β*) and lamin A/C (anti‐lamin C antibody). *N* = 3, and *n* ≥ 100 cells each. b) Importin *β* and lamin A/C fluorescence intensity quantification after analysis of cells (*n* = 20) from each experiment (*N* = 3) where the averaged intensity was determined from 3 different sections of each cell. c) Western blotting of the expression levels of lamin A/C in EA.hy926, A549_0R, and A549_3R cells. d) Western blotting of the levels of importin *β* in EA.hy926, A549_0R, and A549_3R cells. Data are shown as the mean ± SEM. Significant statistical differences (*P* < 0.05, one‐way analysis of variance followed by Bonferroni's multiple comparison test) exist between A549_0R‐A549_3R and EA.hy926 but none between A549_0R and A549_3R (*N* ≥ 3). The housekeeping protein GAPDH is used as a loading control. Scale bars are 10 µm each.

**Figure** **3** compares the levels of lamin A/C in non‐cancer to cancer cells, using immunofluorescence staining and western blotting, and shows that the expression levels of lamins A/C are substantially lowered in cancer cells.

Analogous to our observations with human lung cancer cells, previous publications demonstrate that the expression levels of lamins A/C are markedly reduced in diverse cancer types, which makes lamins A/C a valuable diagnostic and risk biomarker for breast and colorectal cancers.^[^ ^45^, ^46^, ^47^ ^]^ Reduced levels of lamins A/C result in softening of the cell nucleus.^[^ ^43^ ^]^ For metastasis, cancer cells have to detach from the primary tumor to reach distant organs and spread, generally via narrow gaps between the endothelial linings of the vasculature.^[^ ^20^ ^]^ For this purpose, cancer cells must be able to undergo transient dramatic changes in their overall morphology, as seen in Figure 2. The nucleus is the largest intracellular structure and is up to ten times stiffer than the cytoplasm.^[^ ^20^ ^]^ Pathologically induced softening of the nuclei in malignantly transformed cancer cells, resulting from reduction of lamin A/C levels, serves their deformability and enhances their invasion and extravasation potential.^[^ ^20^ ^]^ Our recent study reveals that the physiological stringency of the NPC barrier in non‐cancer cells has a profound impact on the crucial maintenance of nuclear mechanics, which is discussed later. We therefore assume that the parallel pathological reduction of both the NPC barrier stringency and lamin A/C expression levels in malignantly transformed lung cancer cells act synergistically to drive nuclear mechanopathology and enhance cancer metastasis. Indeed, nuclear mechanopathology and resulting dramatic changes in cancer cell morphology and pathophysiology serve as powerful diagnostic tool for the grade of malignancy.^[^ ^24^ ^]^

Another observation made in Figure 3 regards the apparently increased importin ß activity in cancer compared to non‐cancer cells, as supported by findings from immunofluorescence staining and Western blot investigations. This observation lends further support to increased NPC density in cancer cells and may indicate elevated nucleocytoplasmic exchange rates of material. Receptor‐mediated transport of diverse regulatory proteins and newly synthesized ribonucleoproteins between the cytosol and the nucleus is critical for cancer cells homeostasis and survival. Having observed a correlation between the NPC barrier and malignant transformation of non‐small human lung cancer cells, we set out to study the potential impact of pharmacological modulation of the NPC barrier function on cancer cells migration and motility, using NPC barrier breakers. Cancer cells master the controlled manipulation of cell physiology. We hypothesized that lung cancer cells may induce a weakening of the NPC barrier stringency to a well‐balanced level that is just low as absolutely required to promote their pathological transformation and ensure their maintenance. Pharmacological exacerbation of the weakening beyond a “soft spot” may tip the balance and negatively impact lung cancer cells.

At concentrations applied in the present work, the utilized compounds do not dissociate FG‐Nups from the NPC barrier but interfere with their mutual bonds and consequently relax the barrier, as reported in our recent publications.^[^ ^48^, ^49^ ^]^ We used time‐lapse video microscopy to observe highly metastatic A549_3R cells over a time interval of 10 h whereby they were exposed to 2% (m/v) of either NPC barrier breakers 1,2‐trans‐Cyclohexanediol (1,2‐TCHD), 1,6‐Hexanediol (1,6‐HD), in combination (1% each), or their negative controls 1,4‐Cyclohexanediol (1,4‐CHD) and 1,2,3‐Hexanetriol (1,2,3‐HT), respectively.

**Figure 4:**
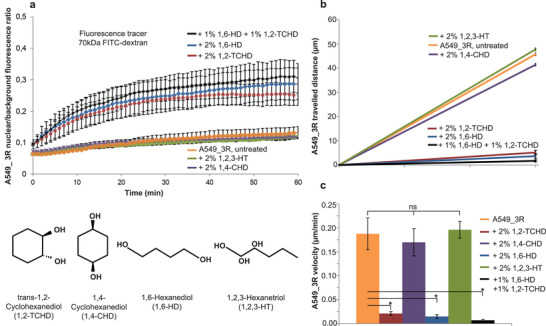
NPC barrier breakers compromise the migratory behavior of highly metastatic lung cancer cells (A549_3R). a) Kinetic profiles of a 70 kDa fluorescent tracer influx into the nuclei of A549_3R cells following cell exposure to NPC barrier breakers 1,6‐Hexanediol (1,6‐HD), 1,2‐trans‐Cyclohexanediol (1,2‐TCHD), or their negative controls 1,4‐Cyclohexanediol (1,4‐CHD) and 1,2,3‐Hexanetriol (1,2,3‐HT), respectively (*N* = 5, and more than 100 cells analyzed in each condition. Data are shown as the mean ± SEM. Statistically significant differences exist between NPC barrier breakers and their negative controls, *P* < 0.05, Student's *t*‐test). b,c) 2D cell migration experiments show that the inhibitory effects of the barrier breakers on cell migration correlate with their ability to disrupt the nucleocytoplasmic permeability barrier (*N* ≥ 3, asterisks indicate significant statistical differences, *P* < 0.05, Student's *t*‐test).

As seen in **Figure** **4**, the NPC barrier breakers 1,6‐HD and 1,2‐TCHD immediately stop the migratory activity of A549_3R cells, in opposite to their negative controls 1,4‐CHD and 1,2,3‐HT, respectively (Videos S1–S6, Supporting Information). 2D cell migration experiments show that the inhibitory effects of the barrier breakers on cell migration correlate with the degree to which they compromise the stringency of the NPC barrier. A combination of 1,6‐HD and 1,2‐TCHD enables 50% reduction of their individual concentrations, while achieving the highest pharmacological effects on both the weakening of the NPC barrier stringency and the inhibition of the migratory behavior of cells. This clearly demonstrates their synergistic behavior.

The NPC barrier breakers and their negative controls show similar behavior in non‐cancer cells (Figure S1, Supporting Information).

**Figure 5:**
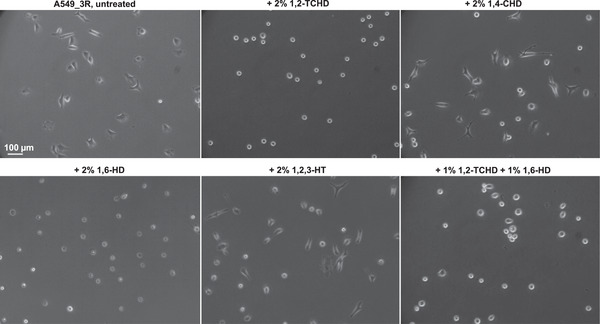
NPC barrier breakers cause drastic overall structural changes of highly metastatic lung cancer cells (A549_3R). Differential interference contrast images collected 10 h after exposure of cells to the NPC barrier breakers 1,2‐trans‐Cyclohexanediol (1,2‐TCHD), 1,6‐Hexanediol (1,6‐HD), or their negative controls 1,4‐Cyclohexanediol (1,4‐CHD) and 1,2,3‐Hexanetriol (1,2,3‐HT), respectively.

The disruption of the NPC barrier stringency is associated with immediate drastic overall structural changes of the cells, which round up and appear to lose traction as shown in **Figure** **5** (Videos S1–S6, Supporting Information).

Analysis of the resulting structural index of A549_3R cells, following 10 h of exposure to NPC barrier breakers and their negative controls is summarized in **Table** **1**.

**Table 1:** Structural index analysis of A549_3R cells 10 h after exposure to NPC barrier breakers and their negative controls

Structural index at 10 h	Untreated A549_3R	+ 2% 1,2‐TCHD	+ 2% 1,4‐CHD	+ 2% 1,6‐HD	+ 2% 1,2,3‐HT	+ 1% 1,2‐TCHD + 1% 1,6‐HD
Mean± SEM	0.41 ± 0.08	0.95 ± 0.04	0.43 ± 0.07	0.96 ± 0.03	0.45 ± 0.06	0.95 ± 0.08

Table 1 Summary of the structural index analysis of highly metastatic lung cancer cells (A549_3R) after 10 h of exposure to the NPC barrier breakers 1,2‐trans‐Cyclohexanediol (1,2‐TCHD), 1,6‐Hexanediol (1,6‐HD), or their negative controls 1,4‐Cyclohexanediol (1,4‐CHD) and 1,2,3‐Hexanetriol (1,2,3‐HT), respectively. *N* = 3, at least 100 analyzed cells in each condition. Significant statistical differences exist between all NPC barrier breakers and their negative controls, but none between the barrier breakers separately or in combination (*P* < 0.05, Student's *t*‐test).

The two compounds 1,2‐TCHD and 1,6‐HD seem to possess necessary chemical and pharmacological properties to act so strongly on the NPC barrier. Their negative controls, 1,4‐CHD and 1,2,3‐HT, respectively, possess similar chemical structures, and yet fail to alter the NPC barrier, migration, motility, or structure of cancer cells.

Figure S2, Supporting Information summarizes LD50 (median lethal dose) measurements following 1 h exposure of non‐cancer (EA.hy926) and cancer cells (A549_0R and A549_3R) to progressively increasing concentrations of the aforementioned NPC barrier breakers and their negative controls.

Summary of the observed effects of NPC barrier breakers leads us to conclude that the extent of migration inhibition as well as the overall structural changes of cells correlate with the degree of the individual compounds to which they compromise the stringency of the NPC permeability. We know from our previous publications that the NPC barrier breakers utilized in the present work at specified concentrations, do not dissociate FG‐Nups from the NPC barrier but rather disrupt their crucial interaction with each other and consequently prevent their selective barrier function.^[^ ^48^, ^49^ ^]^ How can interference with the NPC barrier have such profound impact on the overall structural and dynamical properties of cells? The NPCs are turnstiles of nucleocytoplasmic crosstalk. They are physically and functionally coupled to diverse nucleocytoplasmic structures that span the entire cell.^[^ ^50^, ^51^ ^]^ This intricate nucleocytoplasmic coupling is not only pivotal for maintenance and survival of cells, but also for their overall morphology, mechanics, and migratory activity amongst others.^[^ ^51^, ^52^ ^]^ Hence, the following conceivable consequences may arise from indirect interference with nucleocytoplasmic coupling as a result of direct pharmacological manipulation of the NPC barrier: Structural, mechanical, and functional weakening of the nuclear envelope as a nucleocytoplasmic bridge, alteration in cytoskeletal tension, detachment of sensitive bonds between cells and extracellular matrix (ECM), and disruption of mechanosensing and mechanotransduction.^[^ ^51^, ^52^ ^]^ Given the complexity of such interplay, which remains a subject of intense investigation,^[^ ^51^ ^]^ we prefer to refrain from suggesting possible mechanisms of action as we believe that it would not be possible without relying heavily on speculations. Rather, the findings obtained in the present work, together with closely related findings from our previous works, set the stage for follow‐up studies that will specifically aim at providing mechanistic insights.

We have previously demonstrated that apoptotic cells utilize caspases to dissociate substantial amounts of critical FG‐Nups from the NPC barrier and target the nuclear lamina, which softens the nucleus and renders it particularly vulnerable to mechanical stress.^[^ ^53^, ^54^ ^]^ We therefore postulated the hypothesis that doomed apoptotic cells apply a highly strategic and efficient approach to decisively destabilize nuclear mechanics and ultimately execute the programmed cell death.^[^ ^55^ ^]^ As highlighted in a recent review by Kirby and Lammerding,^[^ ^51^ ^]^ the evidence is emerging that the nucleus including the nuclear envelope plays a key role as a cellular mechanosensor, which is pivotal for cell maintenance and survival. Moreover, pathologically caused nuclear softening gives rise to severe diseases.^[^ ^52^ ^]^ Our recent work reveals that the NPC barrier breaker 1,2‐TCHD, whose potency is similar to that of the other barrier breaker 1,6‐HD as demonstrated in the present work, leads to substantial softening of the cell nucleus.^[^ ^29^ ^]^ We show that slight pharmacological relaxation of the barrier stringency causes remarkable reduction of nuclear resilience to mechanical loads. We also prove that the effects of NPC barrier breakers result primarily from specific interference with the barrier configuration.^[^ ^29^ ^]^ Pharmacologically induced exacerbation of the pathological weakening of the NPC barrier stringency in malignantly transformed cancer cells may compromise their survival ability. Destabilization of nuclear mechanics resulting from NPC barrier breakers may render cancer cells highly sensitive to severe damage during metastatic steps, which pose substantial mechanical stress and require extraordinary high degree of resilience.^[^ ^20^ ^]^ Besides, destabilization of nuclei may impede their critical roles as central hubs for processing mechanical ques from the environment.^[^ ^21^, ^51^ ^]^

**Figure 6:**
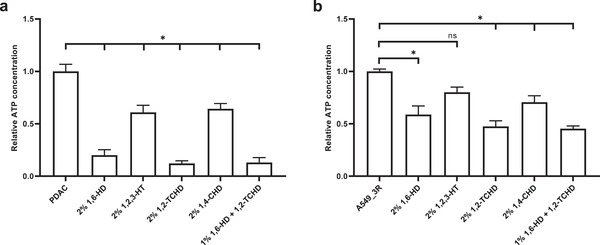
ATP levels in cancer spheroids generated from a) primary pancreatic ductal adenocarcinoma (PDAC)‐derived cells and b) A549_3R cells after 20 h of exposure to the NPC barrier breakers 1,2‐trans‐Cyclohexanediol (1,2‐TCHD), 1,6‐Hexanediol (1,6‐HD), or their negative controls 1,4‐Cyclohexanediol (1,4‐CHD) and 1,2,3‐Hexanetriol (1,2,3‐HT), respectively. Data are shown as the mean ± SEM of 4 individual experiments. Asterisks represent a significant difference compared with the untreated control group, determined using one‐way analysis of variance followed by Bonferroni's multiple comparison test, *P* < 0.05.

To verify that our utilized compounds are not only effective in 2D cell culture models of cancer but also in tumor‐relevant environment, we established 3D cancer models based on our recent publication.^[^ ^56^ ^]^ We tested the compounds for their ability to impact the survival and invasion of cancer cells in cancer spheroids embedded in 3D desmoplastic‐like extracellular matrix (ECM). ATP is crucial for survival of cells and ATP luminescence assays are used as highly sensitive parameter for the prediction and evaluation of tumor sensitivity and resistance to chemotherapeutic drugs.^[^ ^57^ ^]^ Accordingly, we performed ATP luminescence assays to examine the ATP production rates in cancer spheroids embedded in desmoplastic‐like ECM, before and after treatment with the compounds. In addition to A549_3R spheroids and in order to confirm the effectiveness of the compounds in primary cancer cells, we generated spheroids from primary pancreatic ductal adenocarcinoma (PDAC)‐derived cells, isolated from our murine animal model.^[^ ^56^ ^]^ The compounds were added to the mediums of the individual experimental models and compared to compound‐free control cancer spheroids (solvent‐treated), summarized in **Figure** **6**.

As seen in Figure 6 pharmacological treatment is effective in both cancer spheroid models and the effectiveness of the NPC barrier breakers is significantly higher compared to their negative controls. In PDAC spheroids, 2% 1,2,3‐HT (0.61 ± 0.07, *P* < 0.0001) and 2% 1,4‐CHD (0.64 ± 0.05, *P* < 0.0001) decrease total ATP content by around one‐third compared to control (1 ± 0.07). In A549_3R spheroids, 2% 1,4‐CHD is similarly effective at decreasing ATP content (0.64 ± 0.06, *P* < 0.0001), in contrast to 2% 1,2,3‐HT, which does not significantly alter the ATP production (0.84 ± 0.06, *P* = 0.14). In presence of the potent NPC barrier breakers 1,2‐TCHD (2%) and 1,6‐HD (2%) and their combination (1% each), the ATP production in both PDAC (0.12 ± 0.03, *P* < 0. 05; 0.2 ± 0.05, *P* < 0.05; 0.13 ± 0.05, respectively) and A549_3R spheroids (0.47 ± 0.04, *P* < 0.05; 0.52 ± 0.08, *P* < 0.05, 0.46 ± 0.04, *P* < 0.05, respectively) is dramatically decreased compared to control treatment.

The aforementioned findings demonstrate that tumor‐cell derived spheroids embedded into a desmoplastic‐like ECM react to treatment with the compounds by markedly decreasing ATP production. The treatment‐induced strong reduction of the ATP content in cancer spheroids results in their inability to establish sufficient mechanical interaction with the ECM as observed in time‐lapse video microscopy tracing over 20 h (Videos S7–S9, Supporting Information). This observation has direct implications for the survival of tumors and escaping cancer cells. Mechanical interactions between the tumor and its microenvironment are well known to be of fundamental importance for tumor survival. Besides, escape of cancer cells from the tumor and invasion depends crucially on their ability to generate traction forces and contract the ECM in the initial steps preceding the invasion.^[^ ^58^ ^]^ The highly energetic mechanical interactions rely heavily on ATP content in cancer cells. Consequently, the substantial reduction of ATP levels in treated cancer spheroids prevents them from generating sufficient traction forces, which is why they fail to pull on the ECM, in contrast to untreated control PDAC spheroids. The pharmacologically induced reduction of ATP levels may thus be of significant clinical relevance as it may result in decreased tumor spreading in vivo. Interestingly, the applied compounds are differentially effective at decreasing ATP production in A549_3R (≈50% maximal inhibition) and PDAC (≈90% maximal inhibition) spheroids. Here, a likely explanation can be that the basic metabolic profile of these cancers is substantially different. The extreme inhibition of ATP production in primary pancreatic cancer‐derived spheroids is very promising and can further be explained by the fact that these cells did not undergo cell culture‐dependent phenotypic selection.^[^ ^59^ ^]^ The differences in the efficacies of the compounds in the 2D culture as compared to the 3D spheroid systems may arise from the fact that 2D cultures are deficient of the complex tissue architecture and cell‐matrix and cell‐cell interactions which are present in the body.^[^ ^60^ ^]^

It is evident that in particular the NPC barrier breakers have a profound impact on cells, which is further supported by the fact that they give rise to substantial increase of reactive oxygen species (ROS) in cancer cells (Figure S3, Supporting Information). Furthermore, they promote apoptosis as detected in flow cytometric measurements (Figure S4, Supporting Information).

Viability, apoptosis, and necrosis values in EA.hy926 cells after treatment with 2% NPC barrier breakers either separately or in combination, remain in the same range as untreated controls. In contrast, in A549_3R the combination of 1,2‐TCHD and 1,6‐HD promotes apoptosis and causes significant decrease in viability while maintaining low levels of necrosis. This makes the combination an interesting approach for apoptosis‐based treatment of cancer cells, which are known for their apoptosis evasion.^[^ ^61^ ^]^

Currently, there is a limited number of FDA‐approved drugs for cancer therapy that target the apoptotic pathway, many of which target BCL‐2 family members specifically.^[^ ^62^ ^]^ Further research is still needed to understand how NPC barrier breakers affect proteins such as BCL‐2 or NF‐kB. NF‐kB has been shown to mediate the nuclear import of plasmids, for example in lung cancer cells,^[^ ^63^ ^]^ and is a limiting factor in TNF‐oriented apoptosis‐based cancer therapies, due to the systemic inflammation response it triggers.^[^ ^62^ ^]^ Understanding these interactions could help in the development of more efficient approaches for plasmid delivery in gene therapy.

Finally, in the light of the profound impact of the introduced compounds on highly aggressive lung and pancreatic cancers they should be considered for intratumoral application in solid tumor cancers that are fairly common. Their demonstrated instant and substantial effectiveness in 3D scaffold‐based cancer spheroids, which mimic solid tumors, holds promise that they may be able to overcome the harsh tumor barriers, probably owing to their small size and amphiphilic character. The direct delivery of the anticancer drug into the desired place of action has decisive advantages – it achieves instant high local drug concentrations, is more likely to accumulate inside the tumor owing to the prevailing conditions therein, improves drug bioavailability and efficacy, reduces systemic drug concentration, and lowers serious side effects.^[^ ^64^, ^65^ ^]^ The fact that the introduced compounds maintain their effect over at least 10 h of exposure is another significant advantage of particular clinical relevance. For example, the anticancer drug paclitaxel is eliminated to almost 50% within the first 24 h of intravenous administration, and eventually barely 0.5% of the drug is bioavailable at its destined place of action, the tumor site in the lung.^[^ ^65^ ^]^

## 3 Conclusions

The urgently needed improvement of the devastating prognosis of metastatic lung cancer, the major cause of cancer deaths worldwide, requires the refinement of our understanding of the cellular pathophysiological processes that are associated with malignant transformation of lung cancer cells. Despite the overwhelming amount of evidence pointing toward the critical importance of signaling across the nuclear envelope for promoting malignant transformation, the actual involvement of the NPCs is far from being adequately characterized. Our observations demonstrate that the critical ability of the NPCs to exclude substances from the nuclear interior is compromised in lung cancer cells. Apparently, the severity of the defect correlates to some degree with the aggressiveness of the malignant phenotype displayed by the tested cell lines. We also demonstrate the critical importance of an intact selective NPC barrier function for the migration of lung cancer cells. Pharmacological interference instantly disrupts the selective barrier function, which immediately results in prevention of cancer cell migration. The exact cellular physiological mechanisms underlying this inextricable relationship remain to be investigated.

We have recently revealed a profound role of the physiological NPC barrier stringency in maintenance of nuclear mechanics and thus structure.^[^ ^29^ ^]^ When extended to the aforementioned alterations of the factors affecting nucleocytoplasmic transport and the barrier function in cancer, our postulated “ultrafiltration model”^[^ ^29^ ^]^ of nuclear mechanics has several implications for the progression of the process of malignant transformation. An increased expression of the nuclear export factors could shift the balance of macromolecular distribution toward the cytoplasm making the nucleus less resistant toward mechanical deformation. Coupled with the reduced levels of a critical load‐bearing component of the nuclear envelope lamin A/C, this could result in a greatly increased sensitivity of the cancer cell nuclei to physical stresses. These stresses may further exacerbate genomic instability of premalignant cells,^[^ ^66^ ^]^ thus accelerating the rate of malignant transformation. In addition, altered mechanics of the nuclear envelope may interfere with physiological mechanotransduction cascades leading to biochemical signaling which causes active remodeling of the ECM.^[^ ^67^ ^]^

Understanding the ways lung cancer cells exploit this particular element of cellular communication infrastructure may usher in the design of novel anti‐cancer drugs. NPCs are highly conserved among species and so we can imagine that the observations made with aggressive lung and pancreatic cancer models in the present work may be relevant to other types of cancer cells, which should be investigated in future works.

Finally, we would like to propound the idea that NPC barrier breakers may qualify for intratumoral application in solid tumors, pending thorough examination of their drug safety. We can also imagine that a combination of our compounds with well‐established anticancer drugs may have strong synergistic effects. The synergy would not only enable a marked drug dosage reduction, and thus reduction of severe side effects of anticancer drugs, but it may also decisively impact the pharmacological effectiveness and the outcome of the treatment.

## 4 Experimental Section

### Cell Culture

A549 lung adenocarcinoma cells were cultured at 37 °C under 5% CO_2_ in modified Eagle's medium (Invitrogen, Carlsbad, CA), which was supplemented with 10% fetal calf serum. The approach for the generation of highly aggressive non‐small lung cancer cells (A549_3R; R = Selection round) from parental lowly metastatic cancer cells A549_0R was described earlier.^[^ ^68^ ^]^ EA.hy926 endothelial cells were cultured at 37 °C, 5% CO_2_ minimal essential medium (MEM) supplemented with 1% non‐essential amino‐acids, 1% MEM vitamins (Invitrogen), penicillin (100 ug mL^−1^), streptomycin (100 ug mL^−1^), and 10% fetal calf serum (FCS, PAA, Germany). For experimental use, cells were grown to confluence on glass bottom petri dishes (WillCo Wells B. V., Amsterdam, Netherlands).

### Passive Nuclear Pore Permeability Experiments

The diffusion of FITC‐dextran through nuclear pores was tested to evaluate their passive permeability with confocal laser‐scanning microscopy. Cells were cultured on glass bottom petri dishes (WillCo Wells B. V., Amsterdam, Netherlands). Nuclear envelopes were specifically permeabilized following treatment of cells for 5 min with 20 µg mL^−1^ digitonin in transport buffer (TB, 20 mM HEPES, 110 mM K‐Acetate, 5 mM Na‐Acetate, 2 mM Mg‐Acetate, 1 mM EGTA (pH 7.3), 2 mM DTT) containing 200 µg mL^−1^ 10, 20 or 150 kDa FITC‐dextran (Sigma Aldrich, Steinheim, Germany); 150 kDa FITC‐dextran served as an integrity marker of nuclear pores and nuclear envelope. Directly after permeabilization the tested compounds 1,4‐Cyclohexanediol, 1,2‐trans‐Cyclohexanediol, 1,6‐Hexanediol, and 1,2,3‐Hexanetriol (Sigma Aldrich, Steinheim, Germany) were added either separately or in specified combinations at 1 or 2% each. For control experiments, the compounds were replaced with their solvent (water). Confocal microscopy images (objective 63× oil) were taken in the mid‐plane of the nuclei using Leica SP8 confocal laser scanning microscope equipped with hybrid detection system for photon counting (Leica, Wetzlar, Germany) at a rate of one image per minute for 30 min. Subsequently, FITC‐dextran influx dynamics was evaluated by determining the ratio between the intranuclear fluorescence intensity and the extracellular background intensity.

### Western Blot

The lysates of EA.hy96, the A549_0, and A540_3R cells were obtained by adding triton‐lysis buffer (1% Triton X‐100, 150 mm NaCl, 5 mm EDTA, 50 mm Tris‐HCL) and protease inhibitor (cOmplete Mini, Roche Diagnostics GmbH, Mannheim, Germany) to the cells. The protein concentration was detected by the BCA Pierce Protein Assay Kit (Thermo Fisher Scientific Inc., Waltham, MA), and all samples were added with the same amount of protein when tested by Western Blot. Lysate was size‐fractionated with 10% SDS‐Page and blotted onto nitrocellulose membrane (Hybond C‐Extra, Amersham Bioscience, Amersham, UK). The membrane was blocked in 5% skim milk powder solved in TBS (10 mm Tris/HCL, 1,5 m NaCl) with 0.05% Tween 20. The proteins were labeled by using primary and secondary antibodies. First, nitrocellulose membranes were incubated in primary antibodies (Mab414, Covance, 1:1000; Anti‐Nup 98 antibody, Abcam, 1:1000; Anti‐KPNB1 (for importin ß), Abcam 1:5000; Lamin A/C, Cell Signaling Technology, 1:2000; Anti‐*β*‐Actin, Sigma, 1:10 000; Anti‐GAPDH antibody, Abcam, 1:5000) added in blocking buffer overnight. Afterward, the membranes were incubated with the secondary antibodies (Goat anti mouse, DIANOVA, 1:10 000; Anti‐rabbit IgG, Sigma, 1:3000) rarefied in milk for 1 h. By using enzyme substrates (Super Signal West pico/femto, Thermo Scientific), protein bands were visualized by the gel documentation system (ChemiDoc XRS, Bio‐Rad). For quantification, the program ImageJ was used.

### Cell Motility Analysis

EA.hy926 and A549_3R cells were separately seeded on collagen coated culture flasks (containing 1× RPMI, 10 mmol L^−1^ HEPES, 0.02 mg mL^−1^ laminin, 0.04 mg mL^−1^ fibronectin, 0.01 mg mL^−1^ collagen IV, 0.01 mg mL^−1^ collagen III, 0.8 mg mL^−1^ collagen I at pH 7.4) and were left to attach for at least 3 h. Prior to the start of image recording with time‐lapse video microscopy, cells were kept for 10 min in epidermal growth factor (EGF) (1%)‐medium containing the same compounds, combination, and concentration as used in confocal microscopy. In control experiments, the compounds were replaced with their solvent (water). The flasks were transferred to heating chambers (37 °C) on the microscopes (Zeiss Axiovert 40C). The cells were imaged in 600 s intervals for 10 h controlled by HiPic 32 or WASABI software (Hamamatsu). For analysis the Amira Imaging Software and the image processing program ImageJ were used by labeling the cell contours each frame. The cell velocity was calculated from the movement of the cell center per time, the traveled distance was computed by the distance from beginning to the end location and the structure index defines cell morphology (1 = spherical cell, 0 = complex cell).

### Cell Proliferation Assay and LD50 (Median Lethal Dose) Measurements

The CCK‐8 (cell counting kit‐8) assay was used to determine the LD50 values in non‐cancer and cancer cells. The cells were cultured in a 96‐well plate for 24 h at 37 °C, 5% CO_2_. Next, the cells (containing CCK‐8 kit) were exposed for 1 h to progressively increasing concentrations (0.5, 1, 2, 3, 4, 5, 6, 7 and 8%) of the NPC barrier breakers 1,2‐trans‐Cyclohexanediol (1,2‐TCHD), 1,6‐Hexanediol (1,6‐HD), or 1,2‐TCHD and 1,6‐HD in combination, or their negative controls 1,4‐Cyclohexanediol (1,4‐CHD) and 1,2,3‐Hexanetriol (1,2,3‐HT), respectively. The absorbance measurement was performed at 450 nm using the plate reader, Molecular Devices.

### Viability and Apoptosis Measurements with Flow Cytometry

Cells were exposed for 1 h to either 1,2‐TCHD (2%), or 1,6‐HD (2%), or combination of 1,2‐TCHD and 1,6‐HD (1% each), or their negative controls 1,4‐CHD (2%) and 1,2,3‐HT (2%), respectively. After removal of the compounds by several washing steps, a double‐staining was performed with Annexin‐V APCs and propidium iodide (PI) to check for apoptosis and cell viability, respectively. PI was an intercalating dye and stains cells with a compromised plasma membrane as an indicator of necrotic death, measured at 488 nm. Annexin‐V APC was an indicator of apoptosis because it binds the membrane phospholipid phosphatidylserine which was translocated from the inner to the outer leaflet of the plasma membrane during apoptosis, measured at 650–660 nm. 2.5 µL of Annexin‐V APC (Immunotools, Friesoythe, Germany) stock was added to the cells and they were incubated for 15 min in the dark. 300 µL of cell culture medium were added and the cells were placed on ice. Propidium iodide (Merck, Darmstadt, Germany) was added (20 ng mL^−1^) to the cells and the measurements were done immediately using a BD FACS Calibur (BD Biosciences, Erembodegem, Belgium). Data analysis was performed using FlowJo v10 10.5.3 (FlowJo, LLC, Ashland OR) and Microsoft Excel (Microsoft Office 2016).

### Measurement of Intracellular ROS

After treatment of cells for 1 h with NPC barrier breakers and their negative controls at the same concentrations as in the aforementioned experiments, the compounds were washed out and subsequently the intracellular ROS levels were evaluated by using the cellular ROS detection assay kit (ab113851, Abcam), utilizing 2′,7′‐dichlorofluorescin diacetate according to the manufacturer's protocol. The fluorescence measurements were performed using a Fluoroskan II Fluorescent with excitation/emission wavelengths filter: Ex/Em = 485/535 nm.

### Animal Model for Pancreatic Cancer

The animal experiments were conducted with the approval of the local ethics committee for animal care (Landesamt für Natur, Umwelt und Verbraucherschutz Nordrhein‐Westfalen, permit number 81‐02.04.2019.A281). KPfC mice conditionally express mutant K‐Ras (K‐RasG12D) and mutant p53 (p53R172H) in the pancreas under physiological control from the endogenous locus (KRaswt/LSL‐G12D p53wt/LSL‐R172H Pdx1‐Cre+). This was achieved by Lox‐SOP‐Lox (LSL) cassettes that prevent expression of the mutant proteins prior to Cre recombinase‐mediated recombination and consequent excision of the stop cassette.^[^ ^69^, ^70^, ^71^ ^]^

Primary murine pancreatic cancer‐derived cells were isolated as follows. KPfC mice were sacrificed at 24 weeks of age and subsequently, the pancreas was removed. Afterward, pancreata were digested with 1 mg mL^−1^ collagenase P (Sigma Aldrich, Merck KGaA, Darmstadt, Germany) in PBS for 30 min at 37 °C. Then, tissue fragments were centrifuged at 200 g for 5 min RT and cultured in DMEM/HAM F‐12 medium containing 10% FCS and 1% Pen‐Strep in a tissue‐culture dish for 24 h at 37 °C. After 24 h, non‐adherent tissue was washed away 3× with medium and a heterogeneous population of cancer cells, stromal fibroblasts, and immune cells remained in the dish. For the experiments involving spheroids, cells were used from passages 0 to 1.

### Cancer Spheroids Assembly, Matrix Embedding, and Time‐Lapse Video Microscopy

Spheroids of A549 and primary PDAC‐derived cells were created as described previously.^[^ ^56^ ^]^ Briefly, a hanging drop‐based method was used for inducing spheroid formation from 10 000 cells/spheroid, in medium containing 0.25% methylcellulose.

After 48 and 72 h for A549 and PDAC‐derived cells, respectively, spheroids were harvested and embedded in a desmoplastic ECM‐like in vitro polymerized matrix, as described previously.^[^ ^72^ ^]^ The final composition of matrix components were 40 µg mL^−1^ laminin (Sigma Aldrich, Merck KGaA, Darmstadt, Germany), 40 µg mL^−1^ fibronectin (Sigma Aldrich, Merck KGaA, Darmstadt, Germany), 800 µg mL^−1^ collagen I (Corning, New York, NY, USA), 12 µg mL^−1^ collagen III (Corning, New York, NY, USA) and 5.4 µg mL^−1^ collagen IV (BD Biosciences, Heidelberg, Germany). After embedding, spheroids in matrix were further investigated by video microscopy or luminometry.

Time‐lapse video microscopy of spheroids in matrix was performed as described previously.^[^ ^56^ ^]^ First, spheroids in ECM were seeded in 12.5 cm^2^ flasks. After matrix polymerization for 2 h at 37 °C, control medium or medium with target compounds was added to the flasks. After an equilibration period of another 2 h at 37 °C in a 5% CO_2_/air atmosphere, matrix traction of spheroids in the matrix was monitored using time‐lapse video microscopy. Time‐lapse acquisition was recorded in temperature‐controlled chambers (37 °C) with CMOS cameras for 20 h at 5 min intervals using the MicroCamLab 3.1 software (Bresser, Rhede, Germany).

### Measurement of ATP Content in Cancer Spheroids

For measurement of ATP Production, black‐walled 96‐well plates (Corning, New York, NY, USA) were first coated with 1% agarose gel to prevent cell adhesion and spreading at the bottom of the well. Afterward, 50 µL ECM containing one spheroid was applied to each well and left to polymerize for 2 h at 37 °C. Subsequently, control medium or medium with target compounds was added to the wells and spheroids were incubated for 20 h at 37 °C. ATP production of spheroids was measured using the CellTiter‐Glo 3D kit (Promega, Madison, WI, USA) according to manufacturer's protocol. Briefly, 100 µL reagent was added to each well, followed by mechanical disruption of the extracellular matrices containing a spheroid. After 15 min shaking with 200 RPM at RT, total ATP content of the wells was determined using a luminometer (Berthold Technologies, Tristar LB941). The luminescence of each well was background subtracted by luminescence of wells containing ECM without a spheroid.

### Statistical Analysis

Each experimental condition was repeated at least three times. Data are presented as mean values ± standard error of the mean (SEM). Results were considered as statistically significant at the probability level *P* < 0.05. The details regarding the number of experiments and analyzed cells, applied statistical tests and *P* values were specified in the corresponding parts. Statistical tests and graph production were performed using software Origin Pro 9 and GraphPad Prism 9.0.0.

## Conflict of Interest

The authors declare no conflict of interest.

## Supporting information

Supporting Information

Supplemental Video 1

Supplemental Video 2

Supplemental Video 3

Supplemental Video 4

Supplemental Video 5

Supplemental Video 6

Supplemental Video 7

Supplemental Video 8

Supplemental Video 9

## Data Availability

Research data are not shared.
