# Supplementary material for: Impact of the Nuclear Envelope on Malignant Transformation, Motility, and Survival of Lung Cancer Cells
Source: Adv Sci (Weinh). 2021 Oct 17;8(22):2102757. doi: 10.1002/advs.202102757 (PMC8596107; doi:10.1002/advs.202102757)
Supplement: Supplementary file 1 — Supporting Information [file ADVS-8-2102757-s003.pdf]

WILEY-VCH

© 2021 Wiley-VCH GmbH

ADVANCED SCIENCE
Open Access

Supporting Information

for *Adv. Sci.*, DOI: 10.1002/advs.202102757

Impact of the Nuclear Envelope on Malignant Transformation, Motility and Survival of Lung Cancer Cells

Sílvio Terra Stefanello, Isabelle Luchtefeld, Ivan Liashkovich, Zoltan Pethö, Ihab Azzam, Etmar Bulk, Gonzalo Rosso, Lilly Döhlinger, Bettina Hesse, Andrea Oeckinghaus, Victor Shahin**

Supporting Information

Impact of the nuclear envelope on malignant transformation, motility and survival of lung cancer cells

Sílvio Terra Stefanello[1‡], Isabelle Luchtefeld[1‡], Ivan Liashkovich[1], Zoltan Pethö[1], Ihab Azzam[2], Etmar Bulk[1], Gonzalo Rosso[1], Lilly Döhlinger [1], Bettina Hesse[1], Andrea Oeckinghaus[3], Victor Shahin[1*]*

[1] Institute of Physiology II, University of Münster, Robert-Koch-Str. 27b, 48149 Münster, Germany

[2]Institute of Immunology, University of Münster, Röntgen-Str. 21, 48149 Münster, Germany

[3]Institute of Molecular Tumor Biology, University of Münster, Robert-Koch-Str. 43, 48149 Münster, Germany

Dr. Sílvio Terra Stefanello, I. Luchtefeld, Dr. I. Liashkovich, Dr. Z. Pethö, I. Azzam, Dr. E. Bulk, Dr. G. Rosso, L. Döhlinger, Dr. B. Hesse, Dr. A. Oeckinghaus

Institute of Physiology II, University of Münster, Robert-Koch Str. 27b, 48149 Münster, Germany.

‡Equally contributed

***Corresponding Authors:**

Prof. Dr. Victor Shahin and Dr. Ivan Liashkovich

Institute of Physiology II, University of Münster, Robert-Koch Str. 27b

Münster 48149 Münster, Germany

Tel.: +492518355321

E-Mail: shahin@uni-muenster.de E-Mail: liashkov@uni-muenster.de

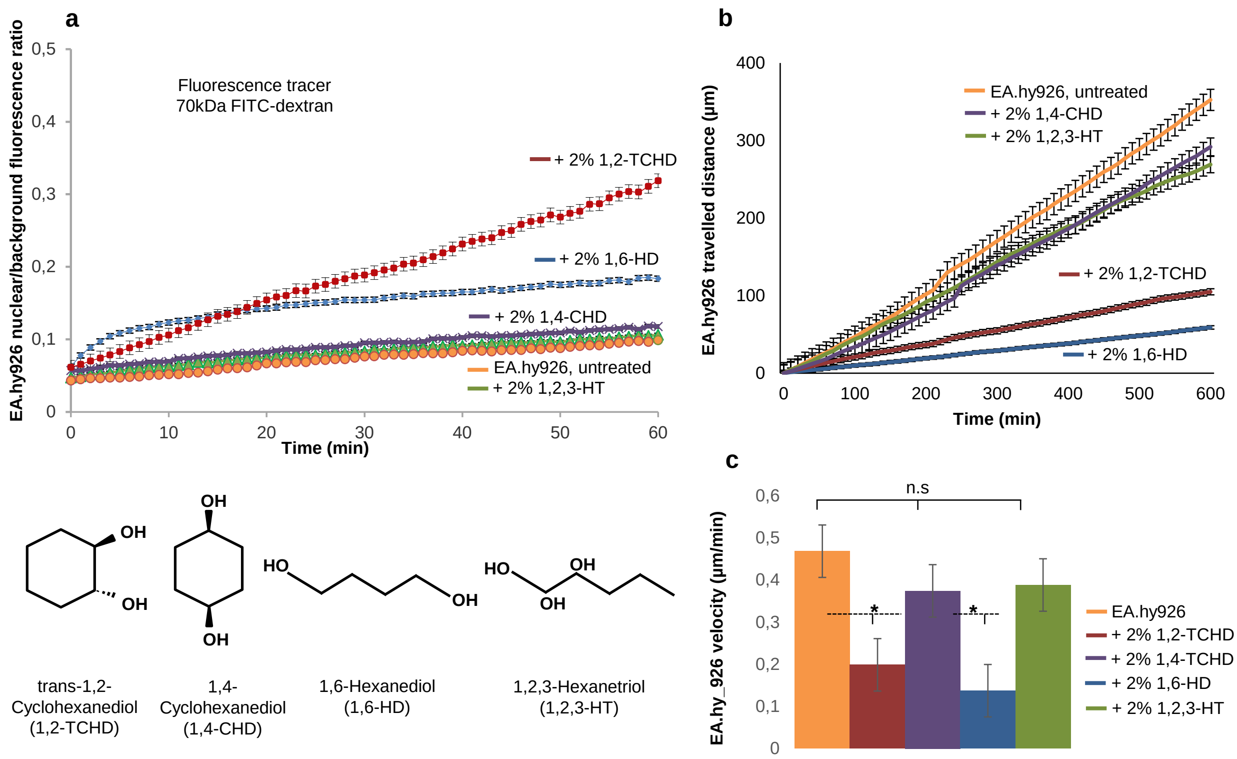

Figure S1. NPC barrier breakers compromise the migratory behavior of non-cancer cells (EA.hy926). a) Kinetic profiles of a 70 kDa fluorescent tracer influx into the nuclei of EA.hy926 cells following exposure to NPC barrier breakers 1,6-Hexanediol (1,6-HD), 1,2-trans-Cyclohexanediol (1,2-TCHD), or their negative controls 1,4-Cyclohexanediol (1,4-CHD) and 1,2,3-Hexanetriol (1,2,3-HT), respectively (N = 3, and more than 100 cells analyzed in each condition. Data are shown as the mean ± SEM. Statistically significant differences exist between NPC barrier breakers and their negative controls, $P < 0.05$, Student's t-test). b) and c) 2D cell migration experiments show that the inhibitory effects of the barrier breakers on cell migration correlate with their ability to disrupt the nucleocytoplasmic permeability barrier (N = 3, asterisks indicate significant statistical differences, $P < 0.05$, Student's t-test).

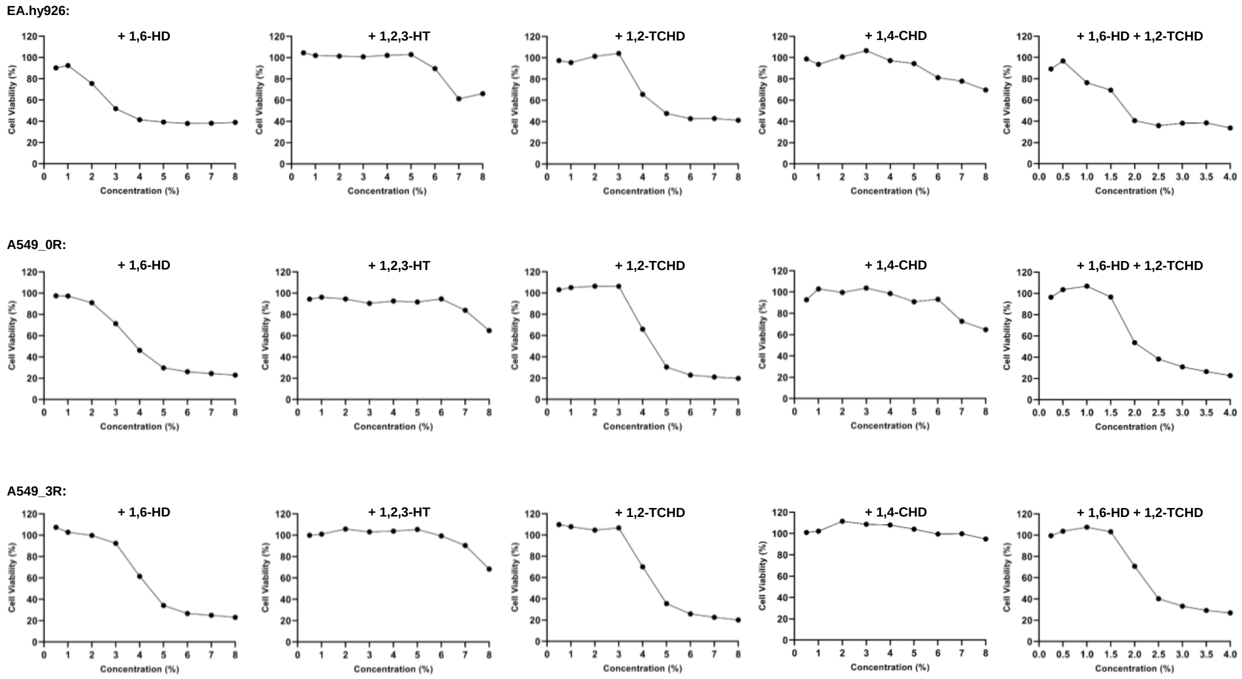

Figure S2. LD50 (median lethal dose) measurements following exposure of non-cancer (EA.hy926) and cancer cells (A549_0R and A549_3R) to NPC barrier breakers and their negative controls. The CCK-8 (cell counting kit-8) assay was used to determine the LD50 values. Cells (in presence of CCK-8) were exposed for 1h to progressively increasing concentrations (0.5, 1, 2, 3, 4, 5, 6, 7 and 8%) of the NPC barrier breakers 1,2-trans-Cyclohexanediol (1,2-TCHD), 1,6-Hexanediol (1,6-HD), or their negative controls 1,4-Cyclohexanediol (1,4-CHD) and 1,2,3-Hexanetriol (1,2,3-HT), respectively.

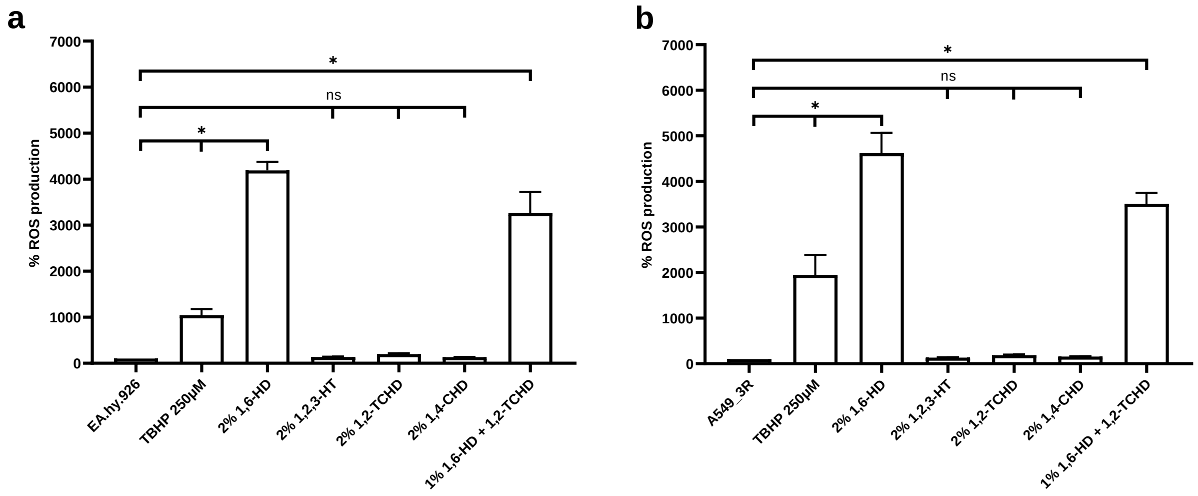

Figure S3. Measurement of reactive oxygen species (ROS) levels in EA.hy926 and A549_3R cells after 1h of exposure to the NPC barrier breakers 1,2-trans-Cyclohexanediol (1,2-TCHD), 1,6-Hexanediol (1,6-HD), or their negative controls 1,4-Cyclohexanediol (1,4-CHD) and 1,2,3-Hexanetriol (1,2,3-HT), respectively. Tert-Butyl hydroperoxide (TBHP) is the positive control. Data are shown as the mean ± SEM of 4 individual experiments. Asterisks represent a significant difference compared with the untreated control group, determined using one-way analysis of variance followed by Bonferroni's multiple comparison test, $P < 0.05$.

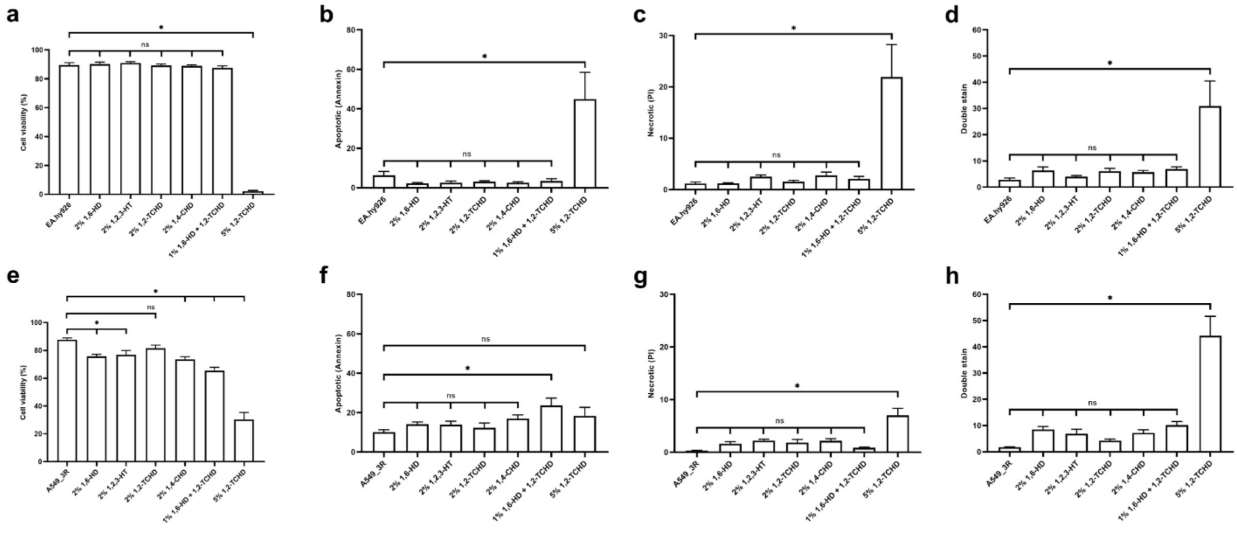

Figure S4. FACS analysis of EA.hy926 and A549_3R cell viability, apoptotic, necrotic and double stain following 1h of exposure to NPC barrier breakers 1,2-trans-Cyclohexanediol (1,2-TCHD), 1,6-Hexanediol (1,6-HD), or their negative controls 1,4-Cyclohexanediol (1,4-CHD) and 1,2,3-Hexanetriol (1,2,3-HT), respectively. Propidium iodide (PI) is an intercalating dye and stains cells with a compromised plasma membrane as an indicator of necrotic death, measured at 488 nm. Annexin-V APC is an indicator of apoptosis because it binds the membrane phospholipid phosphatidylserine which is translocated from the inner to the outer leaflet of the plasma membrane during apoptosis, measured at 650-660 nm. In each experimental condition 30000 cells were analyzed. Data are shown as the mean ± SEM of 4 individual experiments. Asterisks represent a significant difference compared

with the untreated control group, determined using one-way analysis of variance followed by Bonferroni's multiple comparison test, $P < 0.05$.

Supporting videos captions

Video S1. Time-lapse video microscopy of the motility of highly metastatic human lung cancer cells (A549_3R), observed for 10h on 2D collagen matrix.

Video S2. Time-lapse video microscopy of the motility of highly metastatic human lung cancer cells (A549_3R), observed for 10h on 2D collagen matrix, while exposed to the NPC barrier breaker 1,2-trans-Cyclohexanediol (1,2-TCHD, 2%).

Video S3. Time-lapse video microscopy of the motility of highly metastatic human lung cancer cells (A549_3R), observed for 10h on 2D collagen matrix, while exposed to the negative control (1,4-Cyclohexanediol (1,4-CHD), 2%) of the NPC barrier breaker 1,2-trans-Cyclohexanediol.

Video S4. Time-lapse video microscopy of the motility of highly metastatic human lung cancer cells (A549_3R), observed for 10h on 2D collagen matrix, while exposed to the NPC barrier breaker 1,6-Hexanediol (1,6-HD, 2%).

Video S5. Time-lapse video microscopy of the motility of highly metastatic human lung cancer cells (A549_3R), observed for 10h on 2D collagen matrix, while exposed to the negative control (1,2,3-Hexanetriol (1,2,3-HT) 2%) of the NPC barrier breaker 1,6-Hexanediol.

Video S6. Time-lapse video microscopy of the motility of highly metastatic human lung cancer cells (A549_3R), observed for 10h on 2D collagen matrix, while exposed to a combination the two NPC barrier breakers 1,2-trans-Cyclohexanediol (1,2-TCHD) and 1,6-Hexanediol (1,6-HD), 1% each.

Video S7. Time-lapse video microscopy of cancer spheroid generated from primary murine pancreatic cancer-derived cells, observed for 20h while embedded in 3D desmoplastic-like extracellular matrix.

Video S8. Time-lapse video microscopy of cancer spheroid generated from primary murine pancreatic cancer-derived cells, observed for 20h while embedded in 3D desmoplastic-like extracellular matrix in presence of 2% 1,2-trans-Cyclohexanediol.

Video S9. Time-lapse video microscopy of cancer spheroid generated from primary murine pancreatic cancer-derived cells, observed for 20h while embedded in 3D desmoplastic-like extracellular matrix in presence of 2% 1,6-Hexanediol.
